# Supplementary material for: Dietary L-Glu sensing by enteroendocrine cells adjusts food intake via modulating gut PYY/NPF secretion
Source: Nat Commun. 2024 Apr 25;15:3514. doi: 10.1038/s41467-024-47465-4 (PMC11045819; doi:10.1038/s41467-024-47465-4)
Supplement: Supplementary file 1 — Supplementary Information [file 41467_2024_47465_MOESM1_ESM.pdf]

Supplementary Figures：

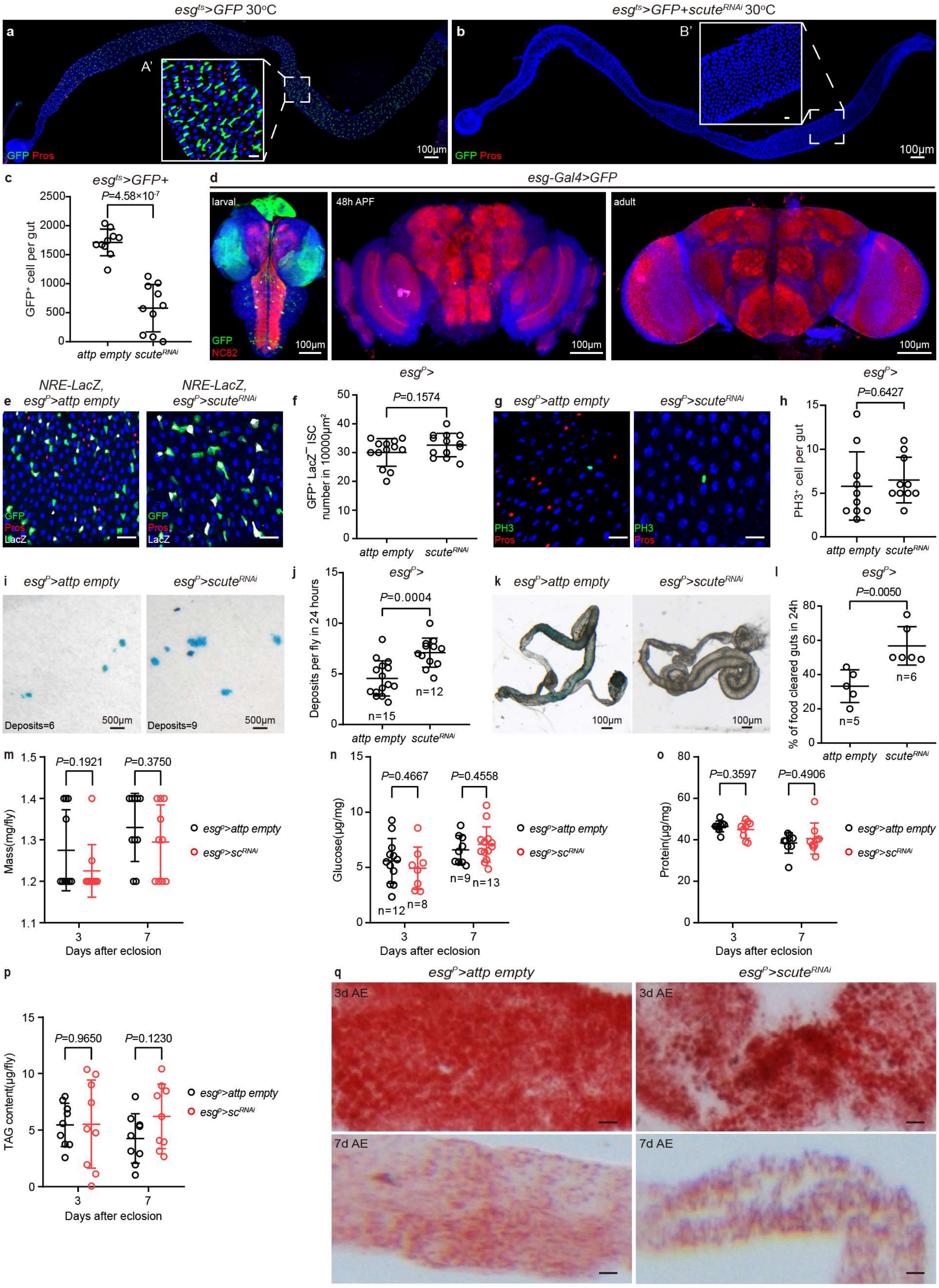

Extended Data Fig. 1 | Loss of EECs enhances the defecation and the speed of gut-clearance but not the division of ISCs and metabolic state.

a, b, Representative images of progenitors (*esg>GFP*, green), EECs (Pros+, red) and DNA (4´, 6-diamidino-2-phenylindole, DAPI, blue in all images) staining in *control* (**a, a'**) and *esg*^{ts}*>GFP+scute*^{RNAi} (**b,**

b') midguts at 30ºC. **a'** and **b'** shows enlarged view of white dashed square in **a** and **b**, respectively. 15 midguts each were examined. **c,** Quantification of GFP^+ progenitor cells of indicated genotypes. n, number of guts. n=10 in each genotype. **d,** The expression pattern of *esg-Gal4>GFP* in larval, 48h APF and adult brains. 16 organs each were examined. **e, f,** Representative images (**e**) and quantification (**f**) of GFP^+, $LacZ^-$ ISCs within 10, 000 μm² in *control* and *NRE-LacZ, esg*^{P}*>GFP+scute*^{RNAi} midguts 3 days after eclosion. n, number of guts. n=13 in each genotype. **g, h,** Representative images (**g**) and quantification (**h**) of $PH3^+$ cells in *control* and *esg*^{P}*>GFP+scute*^{RNAi} midguts. n, number of guts. n=10 in each genotype. **i, j,** Representative images (**i**) and quantification (**j**) of the defecation of *control* and *esg*^{P}*>scute*^{RNAi} flies. n, number of groups (5 flies in each group) performed for defecation quantification. The average deposits per fly was calculated. **k, l,** Representative images (**k**) and percentages (**l**) of midguts cleared blue luminal contents of *control* and *esg*^{P}*>scute*^{RNAi} flies. n, number of groups (5 flies in each group) performed for blue luminal contents clearance quantification. The percentage of fly midguts with empty blue luminal contents were calculated. **m-p,** Mass (**m**), glucose (**n**), protein (**o**) and TAG content (**p**) of *control* and *esg*^{P} *>scute*^{RNAi} flies at 3 and 7 d AE. Each genotype corresponded to 10 (**m**), 9 (**o, p**) samples of 10 (**m**), 20 (**o**) and 10 (**p**) flies each. **q,** Oil Red O staining of *control* and *esg*^{P} *>scute*^{RNAi} midguts at 3 and 7 d AE. 20 midguts each were examined. Data are represented as mean ± SD. Significance was determined using two-sided unpaired *t*-test (**c, f, h, j, l-p**). Source data are provided as a Source Data file. Scale bars, 20 μm except where otherwise specified.

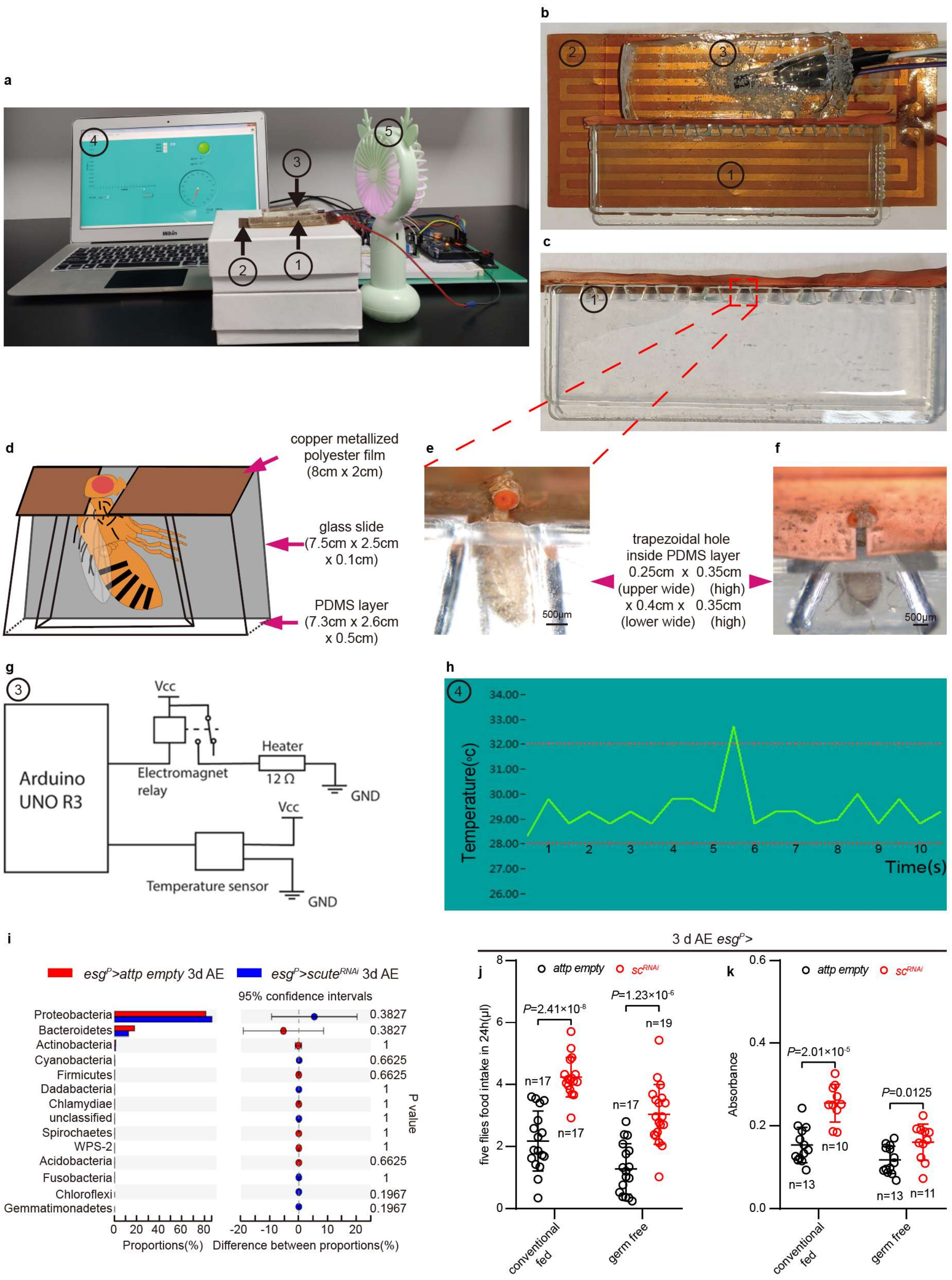

Extended Data Fig. 2 | The temperature control device and gut microbiota has no effect on the rise in food intake due to EEC loss.

a-h, The constitution of temperature control device. **a,** Assembled temperature control device. **b**, The removable fly-placing pad (①), the heating element (②) and the temperature sensor (③). **c**, The removable fly-placing pad. **d**, The cartoon diagram of **e** and **f**. **e, f,** Details of the single fly-placing unit in **c** at different angles. **g**, Diagram of the control circuit. **h**, The user interface of the temperature control software for temperature monitoring. **i,** Microbiota composition of *control* and *esg*^{P}*>scute*^{RNAi} midguts at 3 d AE. **j,** Food intake of *control* and *esg*^{P}*>scute*^{RNAi} flies at 3 d AE under conventional fed and germ free

conditions. **k,** Food consumption of *control* and *esg*^{P}>*scute*^{RNAi} flies at 3 d AE under conventional fed and germ free conditions measured using the dye-based food intake measurement. Data are represented as mean ± SD. Significance was determined using two-sided unpaired *t*-test (**j, k**). n, number of groups performed for quantification of food intake (5 flies in each group) (**j**), or number of groups (20 flies in each group) performed for quantification of food consumption (**k**). Source data are provided as a Source Data file. Scale bars, 500 µm.

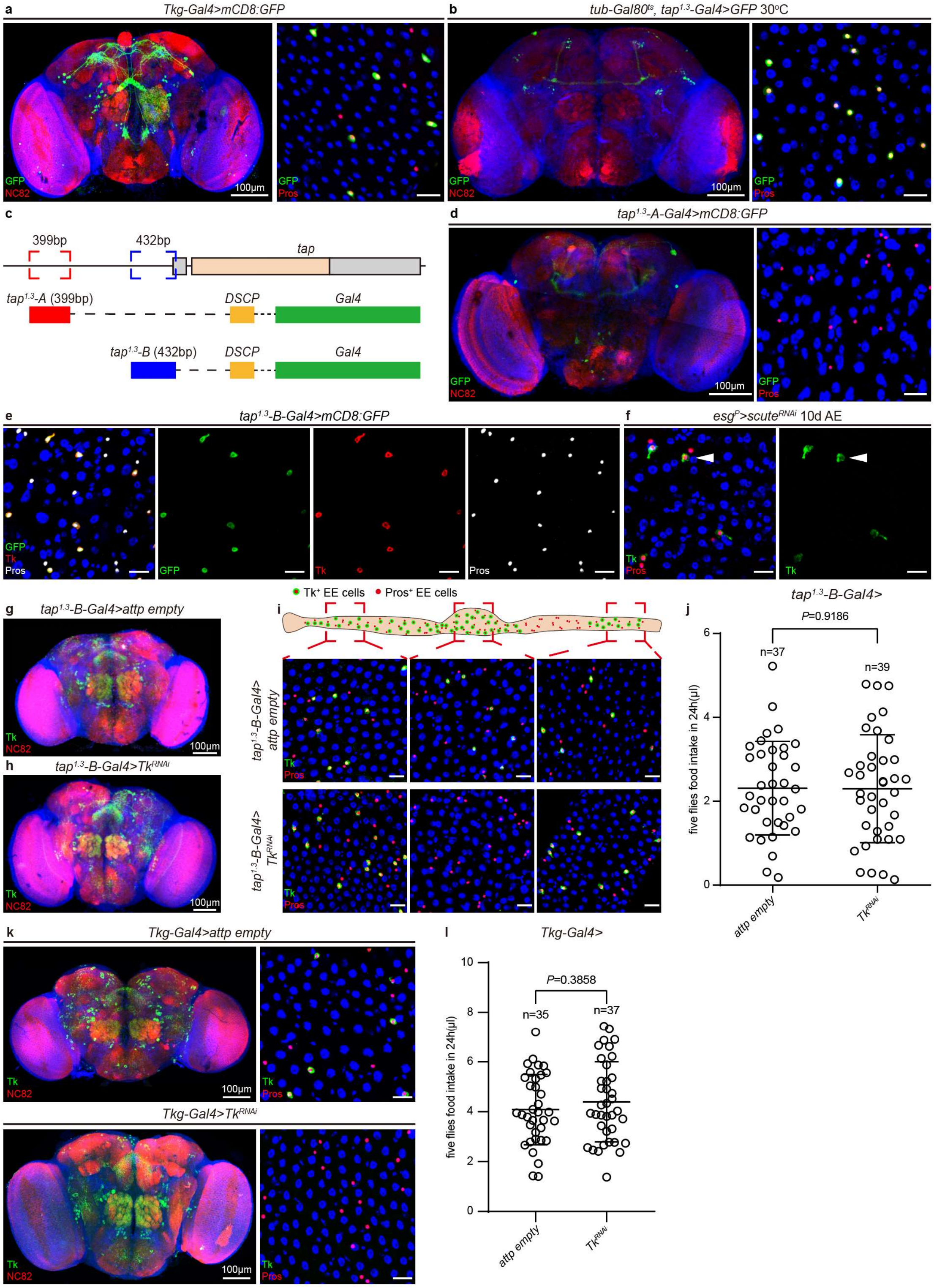

Extended Data Fig. 3 | Generation of an EEC specific driver and EEC-derived Tk has no affect on food intake.

a, The expression pattern of *Tkg-Gal4>mCD8:GFP*, which was expressed in both brain and EECs. 15 flies were examined. **b,** The expression pattern of *tap$^{1.3}$-Gal4>GFP*, which was expressed in both brain and EECs. 15 flies were examined. **c,** Schematic representation of the construction of *tap$^{1.3}$-A-Gal4* and *tap$^{1.3}$-B-Gal4*. **d,** The expression pattern of *tap$^{1.3}$-A-Gal4>mCD8:GFP*, which was expressed in brain but not in EECs. 17 flies were examined. **e,** *tap$^{1.3}$-B-Gal4>GFP^{+}* cells were co-stained with Tk. 17 midguts were examined. **f,** Emerging EECs were co-stained with Tk in *esg^{P}>scute^{RNAi}* midguts after 10 days recovery. 15 midguts were examined. **g-i,** Tk immunostaining in brain (**g, h**) and midgut (**i**) of *control* and *tap$^{1.3}$-B-Gal4>Tk^{RNAi}* flies. Cartoon diagram in **i** shows the expression pattern of *Tk* in midguts. 21 flies each were examined. **j,** Food intake of *control* and *tap$^{1.3}$-B-Gal4>Tk^{RNAi}* flies. **k,** Tk staining in brain and midgut of *control* and *Tkg-Gal4>Tk^{RNAi}* flies. 21 flies were examined. **l,** Food intake of *control* and *Tkg-Gal4>Tk^{RNAi}* flies. Data are represented as mean ± SD. Significance was determined using two-sided unpaired *t*-test (**j, l**). n, number of groups (5 flies in each group) performed for quantification of food intake (**j, l**). Source data are provided as a Source Data file. Scale bars, 20 μm except where otherwise specified.

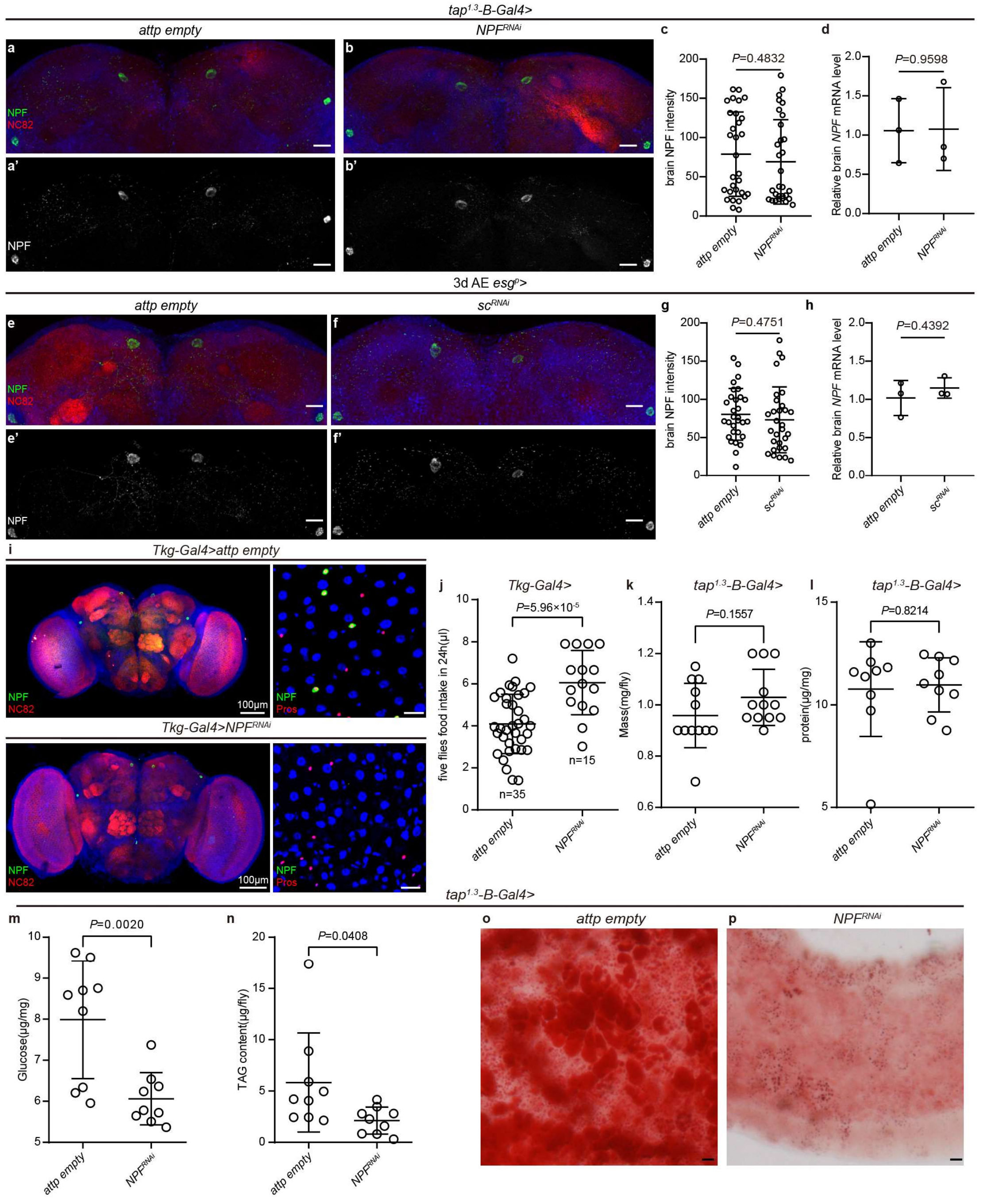

Extended Data Fig. 4 | EEC-derived NPF has no effect on the transcriptional and staining level of NPF in brain, but reduces food intake and increases the glucose and lipid content of flies.

a-c, Representative images (**a**, **b**) and quantification (**c**) of NPF staining in brains of *control* (**a**) and *tap$^{1.3}$-B-Gal4>NPF^{RNAi}* (**b**) flies. n=30 in each group. **d,** Normalized brain *NPF* mRNA levels of *control* and *tap$^{1.3}$-B-Gal4>NPF^{RNAi}* flies by RT–qPCR. Each genotype corresponded to 3 biological replicates of 150 brains each. **e-g,** Representative images (**e, f**) and quantification (**g**) of NPF staining in brains of *control* (**e**) and *esg^{P}>scute^{RNAi}* (**f**) flies at 3 d AE. n=30 in each group. **h,** Normalized brain *NPF* mRNA levels of *control* and *esg^{P}>scute^{RNAi}* flies at 3 d AE by RT–qPCR. Each genotype corresponded to 3 biological replicates of 150 brains each. **i,** NPF staining in brain and midgut of *control* and *Tkg-Gal4>NPF^{RNAi}* flies. 21 flies each were examined. **j,** Food intake of *control* and *Tkg-Gal4>NPF^{RNAi}* flies. **k-n,** Mass (**k**), protein (**l**), glucose (**m**) and TAG content (**n**) of *control* and *tap$^{1.3}$-B-Gal4>NPF^{RNAi}* flies. Each genotype corresponded

to 12 (**k**), 9 (**l-n**) samples of 20 (**k**), 5 (**l**), 20 (**m**) and 10 (**n**) flies each. **o, p,** Oil Red O staining of *control* (**o**) and *tap*$^{1.3}$*-B-Gal4>NPF*^{RNAi} (**p**) midguts. 20 midguts each were examined. Data are represented as mean ± SD. Significance was determined using two-sided unpaired *t*-test (**c, d, g, h, j-n**). n, number of NPF^{+} cells in brain (**c, g**), or number of groups (5 flies in each group) performed for quantification of food intake (**j**). Source data are provided as a Source Data file. Scale bars, 20 μm except where otherwise specified.

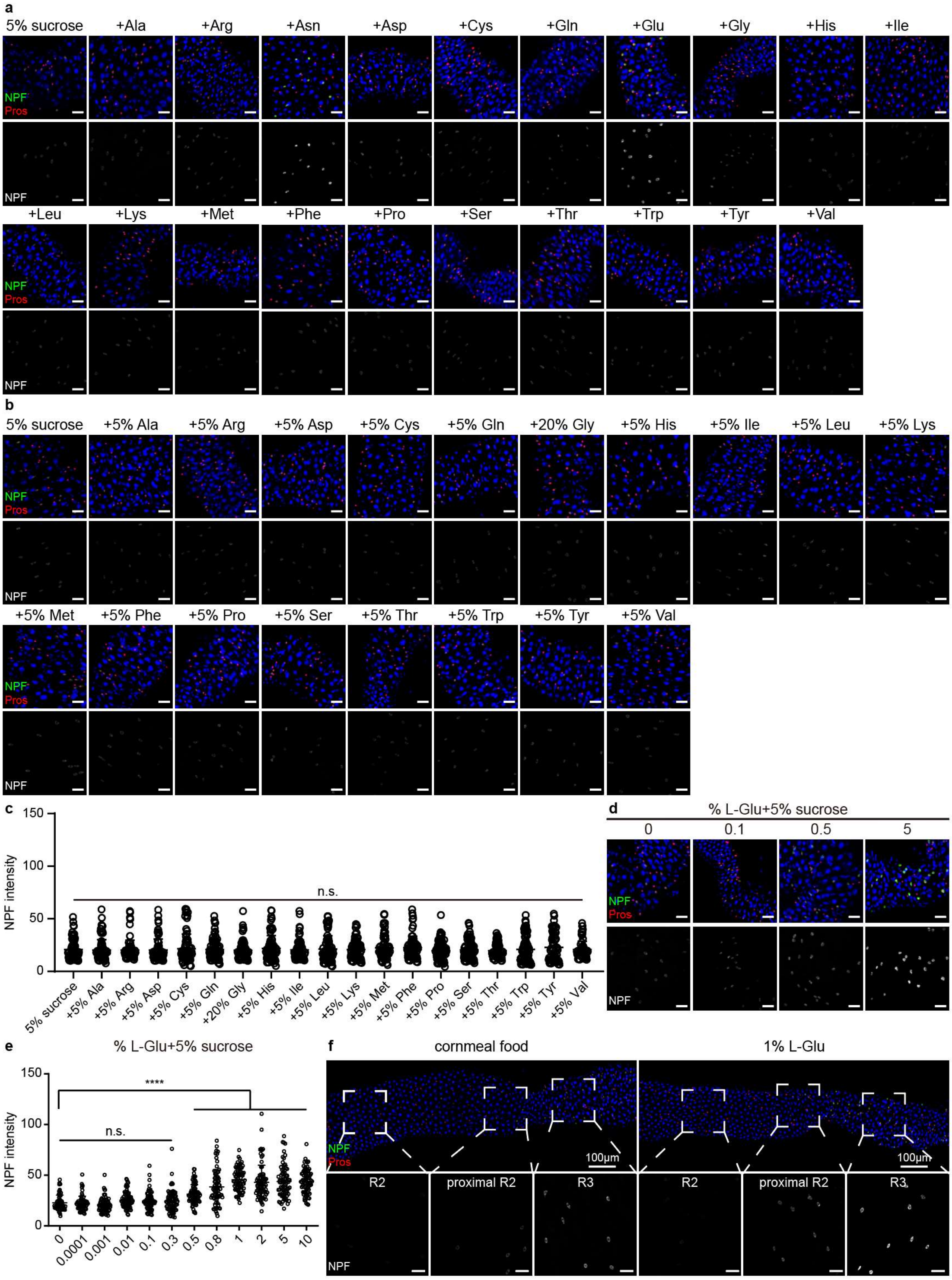

Extended Data Fig. 5 | High concentrations of dietary L-Glu but not another amino acids inhibits

NPF secretion from EECs.

a, Representative images of NPF staining after ingestion of 5% sucrose and 5% sucrose +1% single amino acid. **b, c,** Representative images (**b**) and quantification (**c**) of NPF staining after ingestion of 5% sucrose and 5% sucrose +5% single amino acid except for 20% Gly. n=75 in each group. *p* values are shown in Source Data. **d,** Representative images of NPF staining after ingestion of 5% sucrose and 5% sucrose +0.1%, +0.5% and +5% L-Glu. **e,** Quantification of NPF staining after ingestion of 5% sucrose and 5% sucrose +different concentrations of L-Glu. n=75 in each group. *p* values are shown in Source Data. **f,** NPF staining in EECs of R2, proximal R2 and R3 of midgut under cornmeal food and cornmeal food +1% L-Glu feeding conditions. 27 midguts each were examined. Data are represented as mean ± SD. Significance was determined using two-sided unpaired *t*-test (**c, e**). n.s., non-significant; **** p < 0.0001. n, number of EECs (**c**, **e**). Source data are provided as a Source Data file. Scale bars, 20 μm.

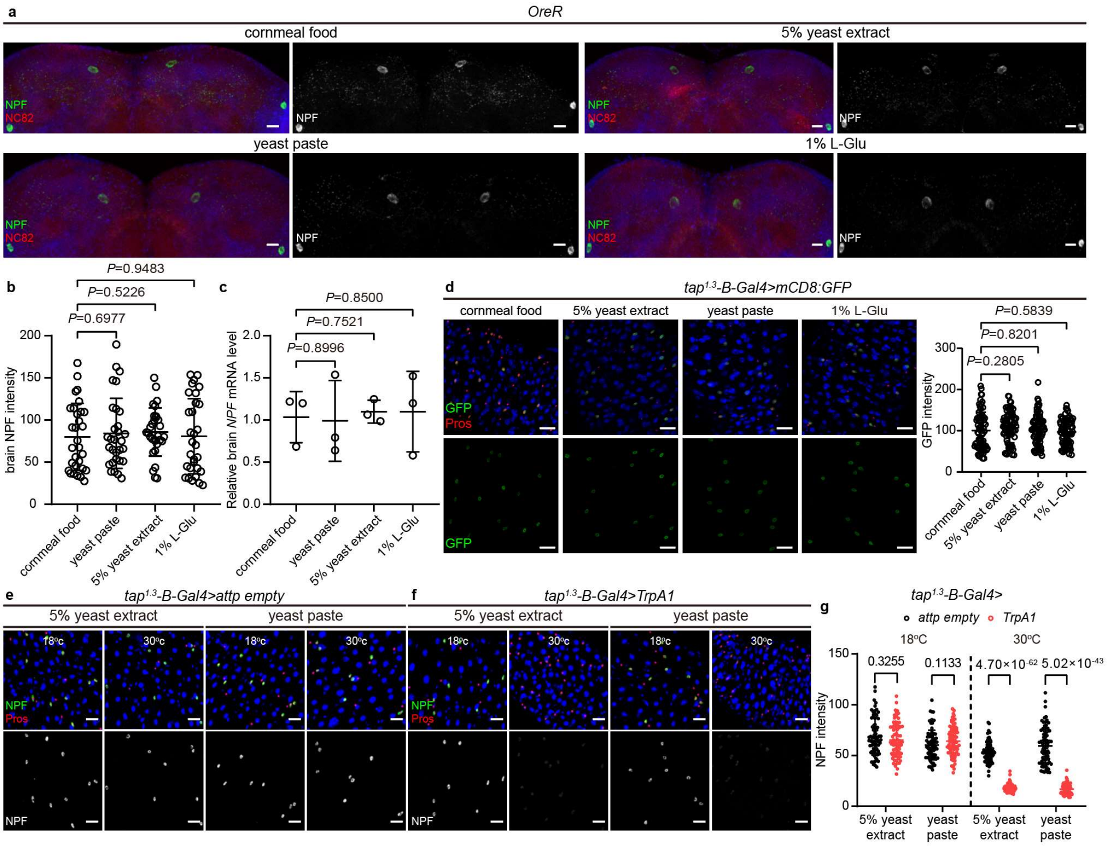

Extended Data Fig. 6 | L-Glu rich diets has no effect on the transcriptional and staining level of NPF in brain, but inhibits NPF secretion from EECs.

a, b, Representative images (**a**) and quantification (**b**) of NPF staining in brains under cornmeal food, 5% yeast extract, yeast paste and 1% L-Glu feeding conditions. n=30 in each group (**b**). **c,** Normalized brain *NPF* mRNA levels under cornmeal food, 5% yeast extract, yeast paste and 1% L-Glu feeding conditions by RT–qPCR. Each genotype corresponded to 3 biological replicates of 150 brains each. **d,** Representative images and quantification of GFP staining in EECs under cornmeal food, 5% yeast extract, yeast paste and 1% L-Glu feeding conditions. n=75 in each group. **e-g,** Under 5% yeast extract or yeast paste feeding conditions, representative images (**e, f**) and quantification (**g**) of NPF staining in EECs of *control* (**e**) and *tap*$^{1.3}$*-B-Gal4>TrpA1* (**f**) flies at 18°C and 30°C. n=75 in each group. *P* values are shown in the figure. Data are represented as mean ± SD. Significance was determined using two-sided unpaired

t-test (**b, c, d, g**). n, number of NPF⁺ cells in brain (**b**), or number of EECs (**d, g**). Source data are provided as a Source Data file. Scale bars, 20 μm.

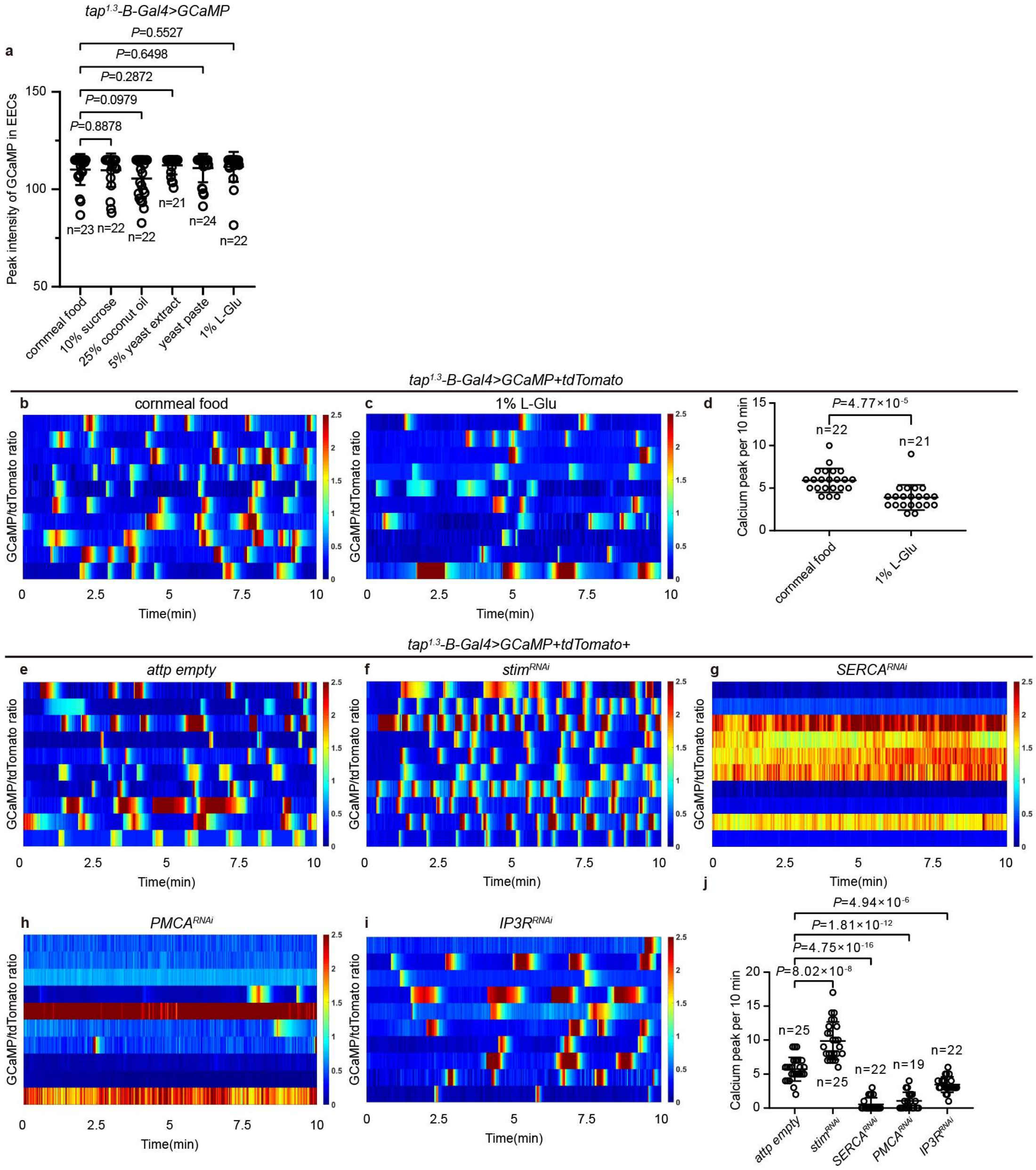

Extended Data Fig. 7 | Dietary L-Glu inhibits calcium oscillations of EECs.

a, The peak Ca^{2+} intensity of GCaMP in EECs after digestion of different food. **b-d,** Representative heatmap records of GCaMP/tdTomato ratio of 10 individual EECs (**b, c**) and quantification of calcium peaks in EECs (**d**) within 10 minutes (300 frames) under cornmeal food (**b**) and 1% L-Glu (**c**) feeding conditions. **e-j,** Representative heatmap records of GCaMP/tdTomato ratio of 10 individual EECs (**e-i**) and quantification of calcium peaks in EECs (**j**) of flies with the indicated genotypes within 10 minutes. Data are represented as mean ± SD. Significance was determined using two-sided unpaired *t*-test (**a, d, j**). n, number of EECs (**a, d, j**). Source data are provided as a Source Data file.

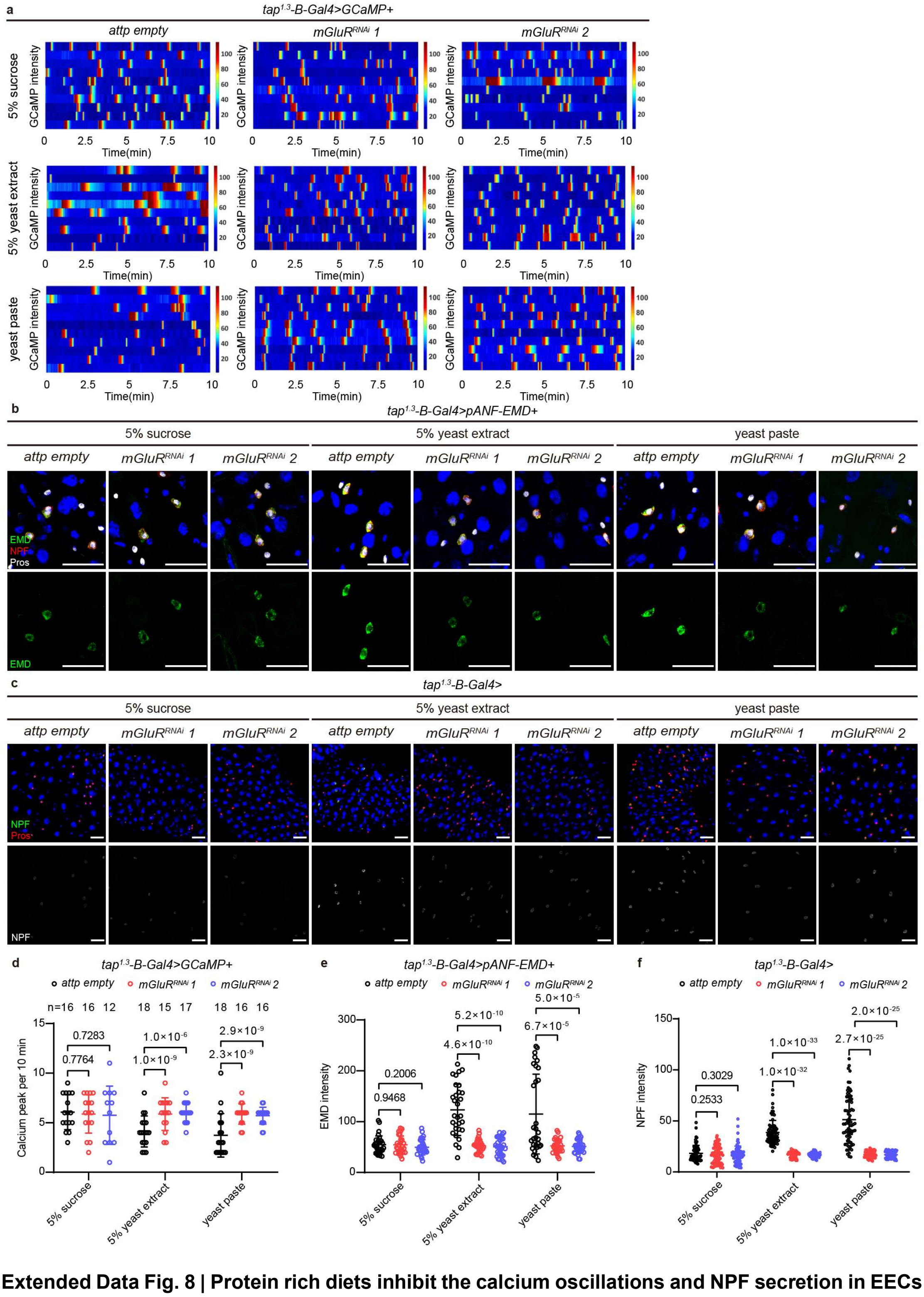

Extended Data Fig. 8 | Protein rich diets inhibit the calcium oscillations and NPF secretion in EECs via mGluR.

a, Under 5% sucrose, 5% yeast extract and yeast paste feeding conditions, representative heatmap records of GCaMP intensity of 10 individual EECs in *control* and *tap$^{1.3}$-B-Gal4>mGluR^{RNAi}* flies within 10 minutes. **b, c,** Under 5% sucrose, 5% yeast extract and yeast paste feeding conditions, representative images of pANF-EMD (**b**) and NPF (**c**) staining in EECs of *control* and *tap$^{1.3}$-B-Gal4>mGluR^{RNAi}* flies. **d,** Quantification of calcium peaks in EECs in *control* and *tap$^{1.3}$-B-Gal4>mGluR^{RNAi}* flies within 10 minutes.

e, f, Under 5% sucrose, 5% yeast extract and yeast paste feeding conditions, quantification of pANF-EMD (**e**) and NPF (**f**) staining in EECs of *control* and *tap^{1.3}-B-Gal4>mGluR^{RNAi}* flies. n=30 (**e**), 75 (**f**) in each group. Data are represented as mean ± SD. Significance was determined using two-sided unpaired *t*-test (**d-f**). *P* values are shown in the figures. n, number of EECs (**d-f**). Source data are provided as a Source Data file. Scale bars, 20μm.

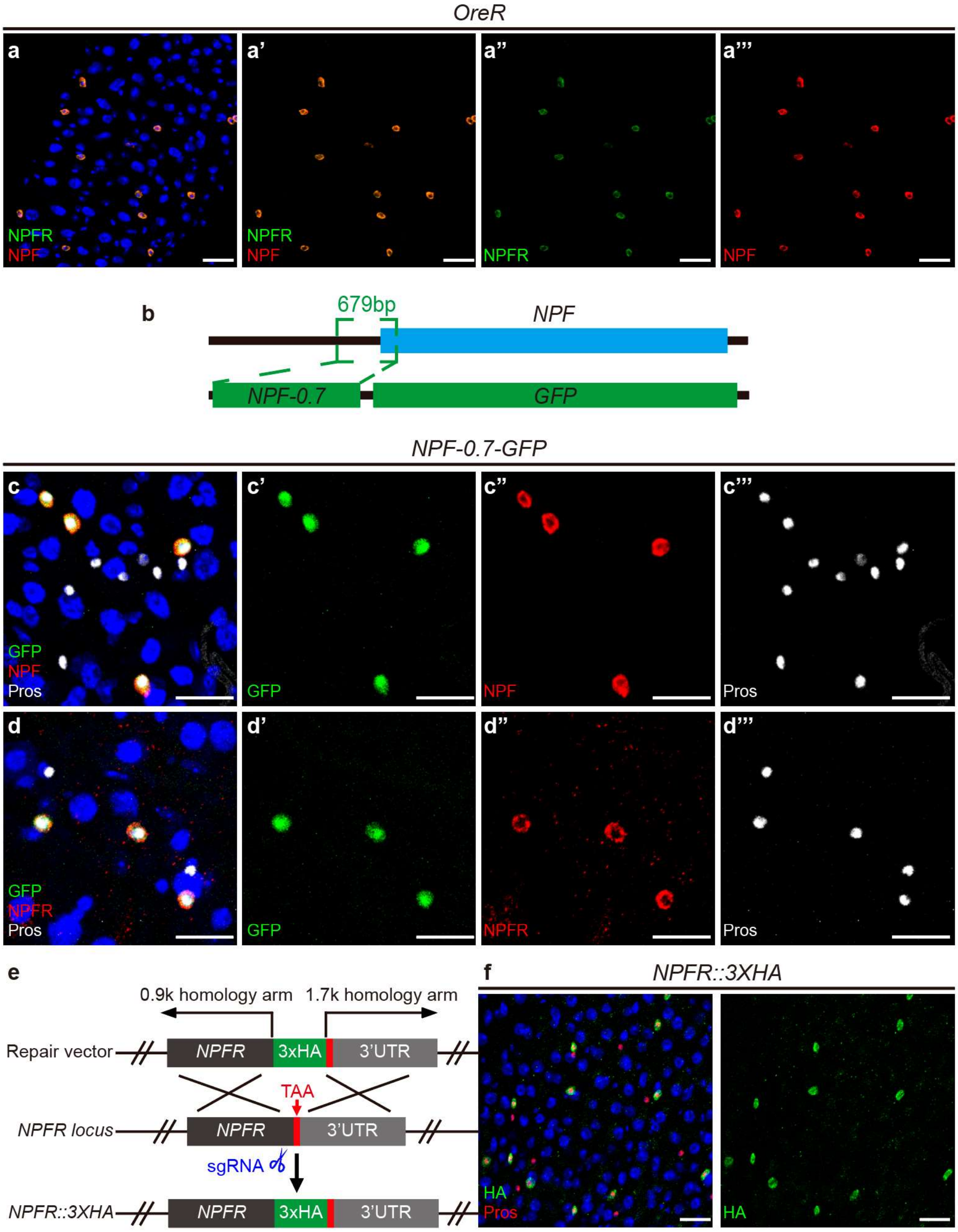

Extended Data Fig. 9 | NPFR is expressed in NPF⁺ EECs.

a, Co-staining of NPFR and NPF shows that NPF and NPFR are expressed in same EECs. 17 midguts were examined. **b,** Schematic representation of the construction of *NPF-0.7-GFP* line. **c, d,** *NPF-0.7-GFP*⁺

cells were co-stained with NPF (**c, c', c", c'"**) and NPFR (**d, d', d", d'"**). 24 midguts each were examined. **e,** Schematic representation of the construction of *NPFR::3XHA*. One sgRNA was used to efficiently induce *NPFR* 3' DNA homologous recombination. 3XHA was inserted before the NPFR stop codon TAA. **f,** The expression pattern of *NPFR::3XHA* in EECs. 15 midguts were examined. Source data are provided as a Source Data file. Scale bars, 20 μm.

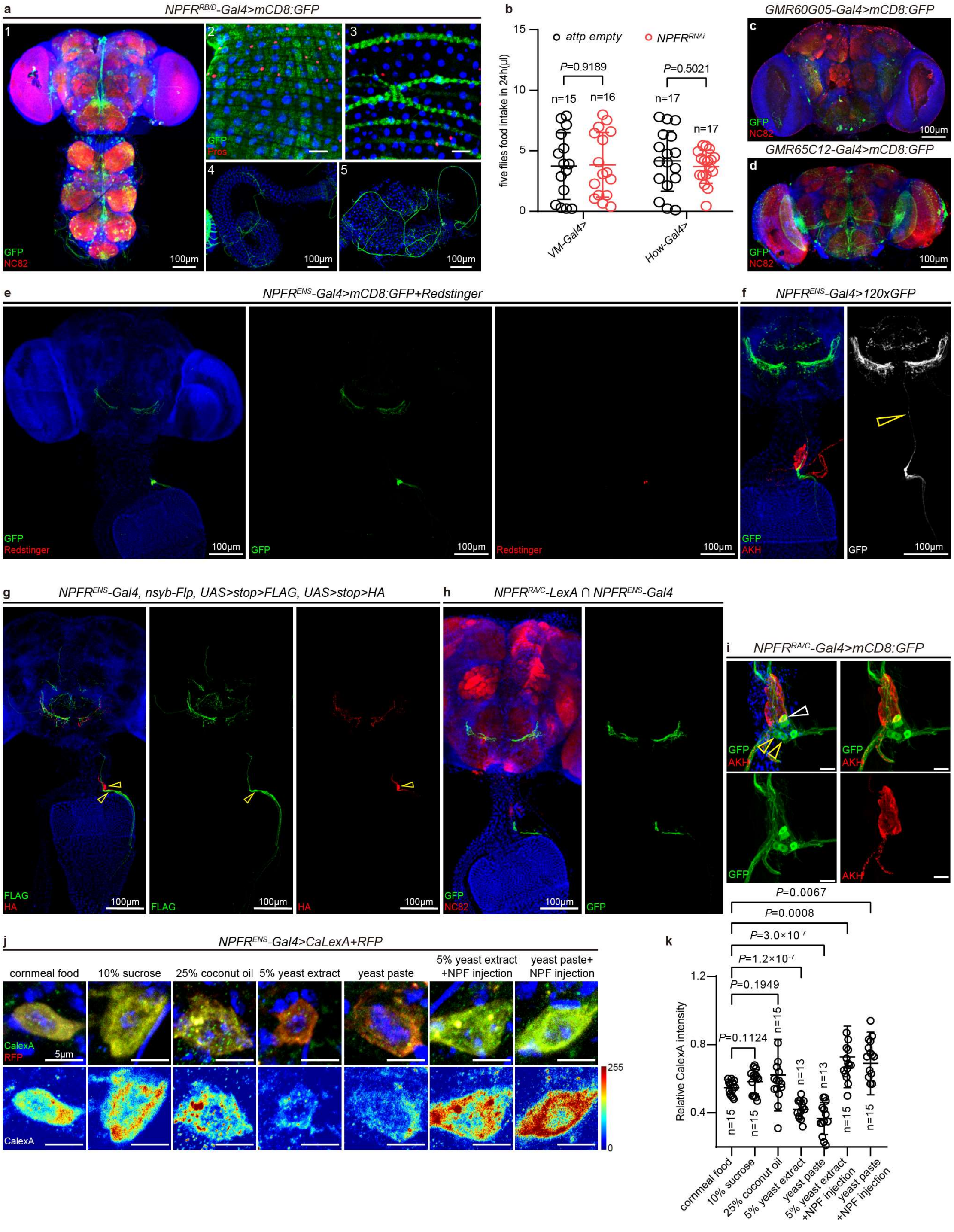

Extended Data Fig. 10 | *NPFR^ENS* neurons locate at HCG and sense NPF secreted from EECs.

a, The expression pattern of *NPFR^RB/D-Gal4>mCD8:GFP* in CNS (1), circular muscle (2) and longitudinal muscle (3) of midgut and neurons in hindgut (4) and rectal ampulla (5). 25 flies were examined. **b,** Food

intake of *control, VM-Gal4>NPRF^RNAi^* and *How-Gal4>NPRF^RNAi^* flies. **c, d,** The expression pattern of *GMR60G05-Gal4* (**c**) and *GMR65C12-Gal4>mCD8:GFP* (**d**) in brains. 24 brains each were examined. **e,** The expression pattern of *$NPFR^{ENS}$-Gal4>mCD8:GFP+Redstinger*. Redstinger shows the location of cell bodies of *$NPFR^{ENS}$-$Gal4^{+}$* neurons. 32 flies were examined. **f,** The expression pattern of *$NPFR^{ENS}$-Gal4>120xGFP*. The yellow arrow head shows the axon of *$NPFR^{ENS}$-$Gal4^{+}$* neurons reaches SEZ of brain. 29 flies were examined. **g,** Stochastic labeling by MultiColor-FlpOut technique (*$NPFR^{ENS}$-Gal4, nsyb-Flp, UAS>stop>FLAG, UAS>stop>HA*) shows that these two *$NPFR^{ENS}$* neurons have similar but diverse projections to the SEZ. Yellow arrowheads point to the location of neuron bodies**.** 26 flies were examined. **h,** Intersectional strategy results show that *$NPFR^{ENS}$-$Gal4^{+}$* neurons are *$NPFR^{RA/C}$-LexA* expressing cells. 25 flies were examined. **i,** Co-staining of AKH and *$NPFR^{RA/C}$-Gal4>mCD8:GFP* shows that while a proportion of *$NPFR>GFP^{+}$* cells have AKH staining (white arrow head), there is still a proportion of *$NPFR>GFP^{+}$* cells without AKH staining (yellow arrow head). 30 flies were examined. **j, k,** Upon indicated manipulations, representative images (**j**) and quantification of relative CaLexA intensity (**k**) in *$NPFR^{ENS}$* neurons. Data are represented as mean ± SD. Significance was determined using two-sided unpaired *t*-test (**b, k**). Source data are provided as a Source Data file. Scale bars, 20 μm unless otherwise specified.

Supplementary Table 1: Fly lines used in this work

Fly line	Stock number	Resource
NPF-0.7-GFP	N/A	This paper
NPFR::3XHA	N/A	This paper
$tap^{1.3}$-A-Gal4	N/A	This paper
$tap^{1.3}$-B-Gal4	N/A	This paper
NRE-Lacz; esg-Gal4,tub-$Gal80^{ts}$, UAS-GFP	N/A	Benjamin Ohlstein, University of Texas Southwestern Medical Center
UAS-$scute^{RNAi}$	THU2205	Tsinghua Fly Center
UAS-attp2 empty	BDSC_36303	BDSC
UAS-attp40 empty	BDSC_36304	BDSC
esg-Gal4	BDSC_93857	Bloomington Drosophila Stock Center (BDSC)
w^{1118}	60000	Vienna Drosophila Resource Center (VDRC)
canton-s	BDSC_36304	BDSC
OreR	BDSC_5	Jianhua Huang, Zhejiang University

tub-Gal480^{ts}*, UAS-GFP; pros*^{v1}*-Gal4*	N/A	Jean-François Ferveur, Université Paris-Sud
UAS-hid	N/A	Benjamin Ohlstein, University of Texas Southwestern Medical Center
Tkg-Gal4	BDSC_93412	Wei Song, Wuhan University
tap$^{1.3}$*-Gal4*	BDSC_46377	BDSC
UAS-nls-GFP	BDSC_4776	BDSC
UAS-Tk^{RNAi}	THU2022	Tsinghua Fly Center
UAS-TNT-imp	BDSC_28841	Zhihua Liu, Hubei University
UAS-TNT-G2	BDSC_28838	Zhihua Liu, Hubei University
UAS-TrpA1	BDSC_26263	Yufeng Pan, Southeast University
UAS-NPF^{RNAi}	THU2569	Tsinghua Fly Center
NPF^{1}	N/A	Shu Kondo, Tokyo University of Science
NPF^{null}	BDSC_84549	Yi Rao, NIBS
UAS-NPF	N/A	Todd Schlenke, University of Arizona
UAS-pANF-EMD	N/A	David L. Deitcher, Cornell University
UAS-GCaMP6f	N/A	Shan Jin, Hubei University
UAS-stim^{RNAi}	THU2581	Tsinghua Fly Center
UAS-SERCA^{RNAi}	THU2107	Tsinghua Fly Center
UAS-PMCA^{RNAi}	THU1887	Tsinghua Fly Center
UAS-IP3R^{RNAi}	TH02220.N	Tsinghua Fly Center

UAS-tdTomato	N/A	Kenneth Irvine, Rutgers University
UAS-CG11155^{RNAi}	THU3285	Tsinghua Fly Center
UAS-clumsy^{RNAi}	BDSC_28351	BDSC
UAS-Ekar^{RNAi}	THU3080	Tsinghua Fly Center
UAS-GluRIA^{RNAi} *1*	TH201500449.S	Tsinghua Fly Center
UAS-GluRIA^{RNAi} *2*	THU2683	Tsinghua Fly Center
UAS-GluRIA^{RNAi} *3*	THU5238	Tsinghua Fly Center
UAS-GluRIB^{RNAi} *1*	THU2758	Tsinghua Fly Center
UAS-GluRIB^{RNAi} *2*	THU5273	Tsinghua Fly Center
UAS-GluRIB^{RNAi} *3*	THU5358	Tsinghua Fly Center
UAS-GluRIIA^{RNAi}	THU2659	Tsinghua Fly Center
UAS-GluRIIB^{RNAi}	THU3089	Tsinghua Fly Center
UAS-GluRIIC^{RNAi}	THU2049	Tsinghua Fly Center
UAS-GluRIID^{RNAi}	THU2151	Tsinghua Fly Center
UAS-GluRIIE^{RNAi}	THU3986	Tsinghua Fly Center
UAS-Grik^{RNAi}	THU3979	Tsinghua Fly Center
UAS-KaiR1D^{RNAi}	THU3982	Tsinghua Fly Center
UAS-mGluR^{RNAi} *1*	THU5288	Tsinghua Fly Center
UAS-mGluR^{RNAi} *2*	THU2115	Tsinghua Fly Center
UAS-mtt^{RNAi} *1*	THU0827	Tsinghua Fly Center
UAS-mtt^{RNAi} *2*	THU5594	Tsinghua Fly Center
UAS-Nmdar1^{RNAi} *1*	THU2118	Tsinghua Fly Center

UAS-Nmdar1^{RNAi} 2	THU5286	Tsinghua Fly Center
UAS-Nmdar1^{RNAi} 3	THU5287	Tsinghua Fly Center
UAS-Nmdar2^{RNAi} 1	THU5240	Tsinghua Fly Center
UAS-Nmdar2^{RNAi} 2	THU5279	Tsinghua Fly Center
UAS-Nmdar2^{RNAi} 3	THU5862	Tsinghua Fly Center
NPFR^{8}	N/A	Shu Kondo, Tokyo University of Science
NPFR^{null}	BDSC_84550	Yi Rao, NIBS
UAS-NPFR^{RNAi}	THU2116	Tsinghua Fly Center
NPFR$^{RA/C}$-Gal4	BDSC_84672	Yi Rao, NIBS
NPFR$^{RB/D}$-Gal4	BDSC_84673	Yi Rao, NIBS
vm-Gal4	BDSC_48547	BDSC
How-Gal4	BDSC_1767	BDSC
GMR60G05-Gal4	BDSC_39259	BDSC
GMR61H06-Gal4	BDSC_39281	BDSC
GMR65C12-Gal4	BDSC_39348	BDSC
GMR60E02-Gal4	BDSC_36304	BDSC
UAS-mCD8:GFP, UAS-Redstinger	N/A	Woo Jae Kim , HIT Center for Life Sciences
20XUAS-6xGFP	BDSC_52262	BDSC
NPFR$^{RA/C}$-LexA	BDSC_84423	Yi Rao, NIBS
nsyb-FlpL;; UAS>stop>FLAG, UAS>stop>HA	BDSC_64087	BDSC
8xLexAop-FlpL, UAS>stop>myr:GFP	N/A	Yufeng Pan, Southeast University
UAS-shi^{ts}	BDSC_66600	BDSC
13xLexAop-myr:GFP, UAS-mCD8:RFP;;10xUAS-CaLexA	N/A	Yufeng Pan, Southeast University
UAS-Denmark, UAS-nSyt:GFP	BDSC_33065	Yufeng Pan, Southeast University
UAS-ChAT^{RNAi} 1	TH02505.N	Tsinghua Fly Center
UAS-ChAT^{RNAi} 2	TH201500313.S	Tsinghua Fly Center

UAS-Ddc^{RNAi}	THU2416	Tsinghua Fly Center
UAS-Vmat^{RNAi}	TH01473.N	Tsinghua Fly Center
UAS-Gad^{RNAi} *1*	TH02214.N	Tsinghua Fly Center
UAS-Gad^{RNAi} *2*	TH201500431.S	Tsinghua Fly Center
UAS-VGAT^{RNAi}	THU4304	Tsinghua Fly Center
UAS-Hdc^{RNAi}	THU2140	Tsinghua Fly Center
UAS-Tbh^{RNAi} *1*	TH02221.N	Tsinghua Fly Center
UAS-Tbh^{RNAi} *2*	TH201500898.S	Tsinghua Fly Center
UAS-Tdc2^{RNAi}	THU2075	Tsinghua Fly Center
UAS-Trh^{RNAi}	THU2052	Tsinghua Fly Center
UAS-VGlut^{RNAi}	THU2700	Tsinghua Fly Center
Ddc-LexA	BDSC_54218	BDSC
13xLexAop-myr:GFP, UAS-mCD8:RFP	BDSC_54218	Yufeng Pan, Southeast University
UAS-myr:GFP, QUAS-tdTomato (3xHA); trans-tango	N/A	Yufeng Pan, Southeast University

Supplementary Table 2: Oligo sequences used for qPCR

Primer name	Sequence
*Rpl23*_F	GACAACACCGGAGCCAAGAACC
*Rpl23*_R	GTTTGCGCTGCCGAATAACCAC
*NPF*_F	ATGTGCCAAACAATGCGTTGCATC
*NPF*_R	TTAGAATATCTCCTCCTCATTAAAA

Supplementary Table 3. Genotypes in this study.

**Figure**	**Genotype**
Extended Data Fig. 1a	*NRE-LacZ; esg-Gal4, tub-Gal80*^{ts}*, UAS-GFP*
Extended Data Fig. 1b	*NRE-LacZ; esg-Gal4, tub-Gal80*^{ts}*, UAS-GFP; UAS-scute*^{RNAi}
Extended Data Fig.	*NRE-LacZ; esg-Gal4, tub-Gal80*^{ts}*, UAS-GFP; UAS-attp2 empty*

1c	NRE-LacZ; esg-Gal4, tub-Gal80^{ts}, UAS-GFP; UAS-scute^{RNAi}
Extended Data Fig. 1d	esg-Gal4/UAS-mCD8:GFP
Extended Data Fig. 1e-q	NRE-LacZ; esg-Gal4, tub-Gal80^{ts}, UAS-GFP; UAS-attp2 empty<br>NRE-LacZ; esg-Gal4, tub-Gal80^{ts}, UAS-GFP; UAS-scute^{RNAi}
Fig. 1a	NRE-LacZ; esg-Gal4, tub-Gal80^{ts}, UAS-GFP; UAS-scute^{RNAi}
Fig. 1b,c	NRE-LacZ; esg-Gal4, tub-Gal80^{ts}, UAS-GFP; UAS-attp2 empty<br>NRE-LacZ; esg-Gal4, tub-Gal80^{ts}, UAS-GFP; UAS-scute^{RNAi}
Fig. 1d	w^{1118}<br>canton-s<br>OreR<br>NRE-LacZ; esg-Gal4, tub-Gal80^{ts}, UAS-GFP<br>UAS-scute^{RNAi}<br>NRE-LacZ; esg-Gal4, tub-Gal80^{ts}, UAS-GFP; UAS-attp2 empty<br>NRE-LacZ; esg-Gal4, tub-Gal80^{ts}, UAS-GFP; UAS-scute^{RNAi}
Fig. 1e	NRE-LacZ; esg-Gal4, tub-Gal80^{ts}, UAS-GFP; UAS-attp2 empty<br>NRE-LacZ; esg-Gal4, tub-Gal80^{ts}, UAS-GFP; UAS-scute^{RNAi}
Fig. 1f	NRE-LacZ; esg-Gal4, tub-Gal80^{ts}, UAS-GFP; UAS-scute^{RNAi}
Fig. 1g,h	NRE-LacZ; esg-Gal4, tub-Gal80^{ts}, UAS-GFP; UAS-attp2 empty<br>NRE-LacZ; esg-Gal4, tub-Gal80^{ts}, UAS-GFP; UAS-scute^{RNAi}
Fig. 1j-p	UAS-hid/+; tub-Gal80^{ts},UAS-GFP; pros^{v1}-Gal4
Fig. 1p	tub-Gal80^{ts},UAS-GFP; pros^{v1}-Gal4/UAS-attp2 empty<br>UAS-hid/+; tub-Gal80^{ts},UAS-GFP; pros^{v1}-Gal4
Extended Data Fig. 2i	NRE-LacZ; esg-Gal4, tub-Gal80^{ts}, UAS-GFP; UAS-attp2 empty<br>NRE-LacZ; esg-Gal4, tub-Gal80^{ts}, UAS-GFP; UAS-scute^{RNAi}
Extended Data Fig. 2j,k	NRE-LacZ; esg-Gal4, tub-Gal80^{ts}, UAS-GFP; UAS-attp2 empty<br>NRE-LacZ; esg-Gal4, tub-Gal80^{ts}, UAS-GFP; UAS-scute^{RNAi}
Extended Data Fig. 3a	UAS-mCD8:GFP; Tkg-Gal4
Extended Data Fig. 3b	tub-Gal80^{ts}, UAS-nls-GFP; tap$^{1.3}$-Gal4
Extended Data Fig. 3d	UAS-mCD8:GFP; tap$^{1.3}$-A-Gal4
Extended Data Fig. 3e	UAS-mCD8:GFP; tap$^{1.3}$-B-Gal4
Extended Data Fig. 3f	NRE-LacZ; esg-Gal4, tub-Gal80^{ts}, UAS-GFP; UAS-scute^{RNAi}
Extended Data Fig. 3g	tap$^{1.3}$-B-Gal4/UAS-attp2 empty
Extended Data Fig. 3h	tap$^{1.3}$-B-Gal4/UAS-Tk^{RNAi}
Extended Data Fig. 3i,j	tap$^{1.3}$-B-Gal4/UAS-attp2 empty<br>tap$^{1.3}$-B-Gal4/UAS-Tk^{RNAi}
Extended Data Fig. 3k,l	Tkg-Gal4/UAS-attp2 empty<br>Tkg-Gal4/UAS-Tk^{RNAi}

Fig. 2a	UAS-mCD8:GFP; tap$^{1.3}$-B-Gal4
Fig. 2b	UAS-TNT-imp; tap$^{1.3}$-B-Gal4<br>UAS-TNT-G2; tap$^{1.3}$-B-Gal4
Fig. 2c	UAS-attp40 empty; tap$^{1.3}$-B-Gal4<br>UAS-TrpA1; tap$^{1.3}$-B-Gal4
Fig. 2d	UAS-mCD8:GFP; tap$^{1.3}$-B-Gal4
Fig. 2e	NRE-LacZ; esg-Gal4, tub-Gal80^{ts}, UAS-GFP; UAS-scute^{RNAi}
Fig. 2f	tap$^{1.3}$-B-Gal4/UAS-attp2 empty
Fig. 2g	tap$^{1.3}$-B-Gal4/UAS-NPF^{RNAi}
Fig. 2h-k, m	tap$^{1.3}$-B-Gal4/UAS-attp2 empty<br>tap$^{1.3}$-B-Gal4/UAS-NPF^{RNAi}
Fig. 2n	NRE-LacZ; esg-Gal4, tub-Gal80^{ts}, UAS-GFP; UAS-attp2 empty<br>NRE-LacZ; esg-Gal4, tub-Gal80^{ts}, UAS-GFP; UAS-scute^{RNAi}
Fig. 2o	NPF^{1}/+<br>NPF^{1}<br>NPF^{null}/+<br>NPF^{null}
Fig. 2p,q	NPF^{1}/+<br>NPF^{1}<br>UAS-NPF; tap$^{1.3}$-B-Gal4, NPF^{1}
Extended Data Fig. 4a-d	tap$^{1.3}$-B-Gal4/UAS-attp2 empty<br>tap$^{1.3}$-B-Gal4/UAS-NPF^{RNAi}
Extended Data Fig. 4e-h	NRE-LacZ; esg-Gal4, tub-Gal80^{ts}, UAS-GFP; UAS-attp2 empty<br>NRE-LacZ; esg-Gal4, tub-Gal80^{ts}, UAS-GFP; UAS-scute^{RNAi}
Extended Data Fig. 4i,j	Tkg-Gal4/UAS-attp2 empty<br>Tkg-Gal4/UAS-NPF^{RNAi}
Extended Data Fig. 4k-n	tap$^{1.3}$-B-Gal4/UAS-attp2 empty<br>tap$^{1.3}$-B-Gal4/UAS-NPF^{RNAi}
Extended Data Fig. 4o	tap$^{1.3}$-B-Gal4/UAS-attp2 empty
Extended Data Fig. 4p	tap$^{1.3}$-B-Gal4/ UAS-NPF^{RNAi}
Fig. 3a-e	OreR
Fig. 3f,g	tap$^{1.3}$-B-Gal4/UAS-pANF-EMD
Fig. 3h-j	UAS-attp40 empty; tap$^{1.3}$-B-Gal4<br>UAS-TrpA1; tap$^{1.3}$-B-Gal4
Fig. 3k	tap$^{1.3}$-B-Gal4/UAS-attp2 empty<br>tap$^{1.3}$-B-Gal4/UAS-NPF^{RNAi}
Fig. 3l	NRE-LacZ; esg-Gal4, tub-Gal80^{ts}, UAS-GFP; UAS-attp2 empty<br>NRE-LacZ; esg-Gal4, tub-Gal80^{ts}, UAS-GFP; UAS-scute^{RNAi}
Fig. 3n	tap$^{1.3}$-B-Gal4/UAS-attp2 empty<br>tap$^{1.3}$-B-Gal4/UAS-NPF^{RNAi}
Extended Data Fig.	OreR

5a-f	
Extended Data Fig. 6a-c	OreR
Extended Data Fig. 6d	UAS-mCD8:GFP; tap$^{1.3}$-B-Gal4
Extended Data Fig. 6e-g	UAS-attp40 empty; tap$^{1.3}$-B-Gal4<br>UAS-TrpA1; tap$^{1.3}$-B-Gal4
Fig. 4a,b	tap$^{1.3}$-B-Gal4/UAS-GCaMP6f
Fig. 4d,e	tap$^{1.3}$-B-Gal4; UAS-GCaMP6f/ UAS-attp2 empty<br>tap$^{1.3}$-B-Gal4; UAS-GCaMP6f/ UAS-stim^{RNAi}<br>tap$^{1.3}$-B-Gal4; UAS-GCaMP6f/ UAS-SERCA^{RNAi}<br>tap$^{1.3}$-B-Gal4; UAS-GCaMP6f UAS-PMCA^{RNAi}<br>tap$^{1.3}$-B-Gal4/ UAS-IP3R^{RNAi}; UAS-GCaMP6f
Fig. 4f,g	tap$^{1.3}$-B-Gal4; UAS-pANF-EMD/ UAS-attp2 empty<br>tap$^{1.3}$-B-Gal4; UAS-pANF-EMD/ UAS-stim^{RNAi}<br>tap$^{1.3}$-B-Gal4; UAS-pANF-EMD/ UAS-SERCA^{RNAi}<br>tap$^{1.3}$-B-Gal4; UAS-pANF-EMD/ UAS-PMCA^{RNAi}<br>tap$^{1.3}$-B-Gal4/ UAS-IP3R^{RNAi}; UAS-pANF-EMD
Fig. 4h-j	tap$^{1.3}$-B-Gal4/ UAS-attp2 empty<br>tap$^{1.3}$-B-Gal4/ UAS-stim^{RNAi}<br>tap$^{1.3}$-B-Gal4/ UAS-SERCA^{RNAi}<br>tap$^{1.3}$-B-Gal4/ UAS-PMCA^{RNAi}<br>UAS-IP3R^{RNAi}; tap$^{1.3}$-B-Gal4
Extended Data Fig. 7a-c	tap$^{1.3}$-B-Gal4, UAS-tdTomato; UAS-GCaMP6f
Extended Data Fig. 7d-i	tap$^{1.3}$-B-Gal4, UAS-tdTomato; UAS-GCaMP6f/ UAS-attp2 empty<br>tap$^{1.3}$-B-Gal4, UAS-tdTomato; UAS-GCaMP6f/ UAS-stim^{RNAi}<br>tap$^{1.3}$-B-Gal4, UAS-tdTomato; UAS-GCaMP6f/ UAS-SERCA^{RNAi}<br>tap$^{1.3}$-B-Gal4, UAS-tdTomato; UAS-GCaMP6f UAS-PMCA^{RNAi}<br>tap$^{1.3}$-B-Gal4, UAS-tdTomato / UAS-IP3R^{RNAi}; UAS-GCaMP6f
Fig. 5a	tap$^{1.3}$-B-Gal4/ UAS-attp2 empty<br>tap$^{1.3}$-B-Gal4/ UAS-CG11155^{RNAi}<br>tap$^{1.3}$-B-Gal4/ UAS-clumsy^{RNAi}<br>tap$^{1.3}$-B-Gal4/ UAS-Ekar^{RNAi}<br>UAS-GluRIA^{RNAi} 1; tap$^{1.3}$-B-Gal4<br>tap$^{1.3}$-B-Gal4/ UAS-GluRIA^{RNAi} 2<br>UAS-GluRIA^{RNAi} 3; tap$^{1.3}$-B-Gal4<br>tap$^{1.3}$-B-Gal4/ UAS-GluRIB^{RNAi} 1<br>UAS-GluRIB^{RNAi} 2; tap$^{1.3}$-B-Gal4<br>tap$^{1.3}$-B-Gal4/ UAS-GluRIB^{RNAi} 3<br>tap$^{1.3}$-B-Gal4/ UAS-GluRIIA^{RNAi}<br>tap$^{1.3}$-B-Gal4/ UAS-GluRIIB^{RNAi}<br>tap$^{1.3}$-B-Gal4/ UAS-GluRIIC^{RNAi}<br>tap$^{1.3}$-B-Gal4/ UAS-GluRIID^{RNAi}

	tap$^{1.3}$-B-Gal4/ UAS-GluRIIE^{RNAi}<br>*tap$^{1.3}$-B-Gal4/ UAS-Grik^{RNAi}*<br>*tap$^{1.3}$-B-Gal4/ UAS-KaiR1D^{RNAi}*<br>*UAS-mGluR^{RNAi} 1; tap$^{1.3}$-B-Gal4*<br>*tap$^{1.3}$-B-Gal4/ UAS-mGluR^{RNAi} 2*<br>*tap$^{1.3}$-B-Gal4/ UAS-mtt^{RNAi} 1*<br>*UAS-mtt^{RNAi} 2; tap$^{1.3}$-B-Gal4*<br>*tap$^{1.3}$-B-Gal4/ UAS-Nmdar1^{RNAi} 1*<br>*tap$^{1.3}$-B-Gal4/ UAS-Nmdar1^{RNAi} 2*<br>*UAS-Nmdar1^{RNAi} 3; tap$^{1.3}$-B-Gal4*<br>*tap$^{1.3}$-B-Gal4/ UAS-Nmdar2^{RNAi} 1*<br>*UAS-Nmdar2^{RNAi} 2; tap$^{1.3}$-B-Gal4*<br>*tap$^{1.3}$-B-Gal4/ UAS-Nmdar2^{RNAi} 3*
Fig. 5b,e	*tap$^{1.3}$-B-Gal4; UAS-GCaMP6f/ UAS-attp2 empty*<br>*tap$^{1.3}$-B-Gal4/ UAS-mGluR^{RNAi} 1; UAS-GCaMP6f*<br>*tap$^{1.3}$-B-Gal4; UAS-GCaMP6f/ UAS-mGluR^{RNAi} 2*
Fig. 5c,f	*tap$^{1.3}$-B-Gal4; UAS-pANF-EMD/ UAS-attp2 empty*<br>*tap$^{1.3}$-B-Gal4/ UAS-mGluR^{RNAi} 1; UAS-pANF-EMD*<br>*tap$^{1.3}$-B-Gal4; UAS-pANF-EMD/ UAS-mGluR^{RNAi} 2*
Fig. 5d,g	*tap$^{1.3}$-B-Gal4/ UAS-attp2 empty*<br>*UAS-mGluR^{RNAi} 1; tap$^{1.3}$-B-Gal4*<br>*tap$^{1.3}$-B-Gal4/ UAS-mGluR^{RNAi} 2*
Extended Data Fig. 8a	*tap$^{1.3}$-B-Gal4/UAS-GCaMP6f*
Extended Data Fig. 8b,e	*tap$^{1.3}$-B-Gal4; UAS-GCaMP6f/ UAS-attp2 empty*<br>*tap$^{1.3}$-B-Gal4/ UAS-mGluR^{RNAi} 1; UAS-GCaMP6f*<br>*tap$^{1.3}$-B-Gal4; UAS-GCaMP6f/ UAS-mGluR^{RNAi} 2*
Extended Data Fig. 8c,f	*tap$^{1.3}$-B-Gal4; UAS-pANF-EMD/ UAS-attp2 empty*<br>*tap$^{1.3}$-B-Gal4/ UAS-mGluR^{RNAi} 1; UAS-pANF-EMD*<br>*tap$^{1.3}$-B-Gal4; UAS-pANF-EMD/ UAS-mGluR^{RNAi} 2*
Extended Data Fig. 8d,g	*tap$^{1.3}$-B-Gal4/ UAS-attp2 empty*<br>*UAS-mGluR^{RNAi} 1; tap$^{1.3}$-B-Gal4*<br>*tap$^{1.3}$-B-Gal4/ UAS-mGluR^{RNAi} 2*
Fig. 6a	*NPFR^{8}/+*<br>*NPFR^{8}*<br>*NPFR^{null}/+*<br>*NPFR^{null}*
Fig. 6b,c	*tap$^{1.3}$-B-Gal4/ UAS-attp2 empty*<br>*tap$^{1.3}$-B-Gal4/ UAS-NPFR^{RNAi}*
Fig. 6d	*UAS-mCD8:GFP; NPFR$^{RA/C}$-Gal4*
Fig. 6e	*UAS-mCD8:GFP; GMR60E02-Gal4*
Fig. 6f	*GMR60E02-Gal4/ UAS-attp2 empty*<br>*GMR60E02-Gal4/ UAS-NPFR^{RNAi}*<br>*UAS-hid;; GMR60E02-Gal4*

Fig. 6g	*GMR60E02-Gal4/ UAS-attp2 empty*<br>*GMR60E02-Gal4/ UAS-shi*^{ts}<br>*UAS-TrpA1; GMR60E02-Gal4*
Fig. 6h,i	*13xLexAop-myr:GFP, UAS-mCD8:RFP;; 10xUAS-CaLexA/GMR60E02-Gal4*
Fig. 6j	*GMR60E02-Gal4/ UAS-attp2 empty*<br>*GMR60E02-Gal4/ UAS-NPFR*^{RNAi}
Fig. 6k	*GMR60E02-Gal4/ UAS-attp2 empty*<br>*GMR60E02-Gal4/ UAS-shi*^{ts}
Fig. 6l	*UAS-attp40 empty; GMR60E02-Gal4*<br>*UAS-TrpA1; GMR60E02-Gal4*
Extended Data Fig. 9a	*OreR*
Extended Data Fig. 9c,d	*NPF-0.7-GFP*
Extended Data Fig. 9f	*NPFR::3xHA*
Extended Data Fig. 10a	*UAS-mCD8:GFP; NPFR*$^{RB/D}$*-Gal4*
Extended Data Fig. 10b	*VM-Gal4/ UAS-attp2 empty*<br>*VM-Gal4/ UAS-NPFR*^{RNAi}<br>*How-Gal4/ UAS-attp2 empty*<br>*How-Gal4/ UAS-NPFR*^{RNAi}
Extended Data Fig. 10c	*UAS-mCD8:GFP; GMR60G05-Gal4*
Extended Data Fig. 10d	*UAS-mCD8:GFP; GMR65C12-Gal4*
Extended Data Fig. 10e	*GMR60E02-Gal4/ UAS-mCD8:GFP, UAS-Redstinger*
Extended Data Fig. 10f	*GMR60E02-Gal4/20XUAS-6xGFP*
Extended Data Fig. 10g	*nsyb-FlpL;;GMR60E02-Gal4/UAS>stop>FLAG, UAS>stop>HA*
Extended Data Fig. 10h	*8xLexAop-FlpL, UAS >stop >myr:GFP; GMR60E02-Gal4/NPFR-RA/C-LexA*
Extended Data Fig. 10i	*UAS-mCD8:GFP; NPFR*$^{RA/C}$*-Gal4*
Extended Data Fig. 10j, k	*13xLexAop-myr:GFP, UAS-mCD8:RFP;; 10xUAS-CaLexA/GMR60E02-Gal4*
Fig. 7a	*GMR60E02-Gal4/ UAS-Denmark, UAS-nSyt:GFP*
Fig. 7b	*GMR60E02-Gal4/ UAS-attp2 empty*<br>*UAS-ChAT*^{RNAi} *1; GMR60E02-Gal4*<br>*UAS-ChAT*^{RNAi} *2; GMR60E02-Gal4*<br>*GMR60E02-Gal4/ UAS-Ddc*^{RNAi}<br>*GMR60E02-Gal4/ UAS-Vmat*^{RNAi}

	UAS-Gad1^{RNAi} 1; GMR60E02-Gal4<br>*UAS-Gad1^{RNAi} 2; GMR60E02-Gal4*<br>*GMR60E02Gal4/ UAS-VGAT^{RNAi}*<br>*GMR60E02-Gal4/ UAS-Hdc^{RNAi}*<br>*UAS-Tbh^{RNAi} 1; GMR60E02-Gal4*<br>*UAS-Tbh^{RNAi} 2; GMR60E02-Gal4*<br>*GMR60E02-Gal4/ UAS-Tdc2^{RNAi}*<br>*GMR60E02-Gal4/ UAS-Trh^{RNAi}*<br>*GMR60E02-Gal4/ UAS-VGlut^{RNAi}*
Fig. 7c	*13xLexAop-myr:GFP, UAS-mCD8:RFP; Ddc-LexA; GMR60E02-Gal4*
Fig. 7d	*UAS-mCD8:GFP; GMR60E02-Gal4*
Fig. 7e	*UAS-myr:GFP, QUAS-tdTomato (3xHA); trans-tango/ GMR60E02-Gal4*
**Supplementary movie**	
movie 1	*tap$^{1.3}$-B-Gal4/UAS-attp2 empty*<br>*tap$^{1.3}$-B-Gal4/UAS-NPF^{RNAi}*
movie 2 and 3	*tap$^{1.3}$-B-Gal4/UAS-GCaMP6f*
movie 4	*tap$^{1.3}$-B-Gal4; UAS-GCaMP6f/ UAS-attp2 empty*
movie 5	*tap$^{1.3}$-B-Gal4; UAS-GCaMP6f/ UAS-stim^{RNAi}*
movie 6	*tap$^{1.3}$-B-Gal4; UAS-GCaMP6f/ UAS-SERCA^{RNAi}*
movie 7	*tap$^{1.3}$-B-Gal4; UAS-GCaMP6f UAS-PMCA^{RNAi}*
