# Supplementary material for: Dietary L-Glu sensing by enteroendocrine cells adjusts food intake via modulating gut PYY/NPF secretion
Source: Nat Commun. 2024 Apr 25;15:3514. doi: 10.1038/s41467-024-47465-4 (PMC11045819; doi:10.1038/s41467-024-47465-4)
Supplement: Supplementary file 3 — Description of Additional Supplementary Files [file 41467_2024_47465_MOESM3_ESM.docx]

**Description of Additional Supplementary Files**

**Supplementary Movie 1 | *NPF* depleted in EECs promotes feeding.**

*Control* (*tap^1.3^-B-Gal4>attp empty*, left) and *tap^1.3^-B-Gal4>NPF^RNAi^* (right) flies aged 3-5 d at 25^o^C were fasted for 36 h before being individually fixed in a 200 µl pipette tip and blocked with cotton. The proboscis was exposed. Flies were then presented with 5 μl of 5% sucrose containing 0.25% (v/v) blue dye liquid food in a glass capillary until they stopped responding to food stimuli for ten serial food stimuli.

**Supplementary Movie 2 | Cytosolic Ca^2+^ oscillation in EECs of flies raised in cornmeal food.**

Guts from *tap^1.3^-B-Gal4>GCaMP6f* flies aged 5 d AE at 25^o^C were dissected and imaged in LIG using a Zeiss LSM 800 confocal microscope. For this and the following videos, a single-layer image of 512x512 pixels (319.45 µm x 319.45 µm) was acquired every second for 10 min at room temperature (25°C) with a pixel time of 1.03 µs and fixed laser power, pinhole and other settings for all time-lapse experiments. *GCaMP6f* emission was recorded at 400-533 nm. The videos were exported uncompressed from ZEN 2.

**Supplementary Movie 3 | L-Glu feeding reduces Ca^2+^ oscillation frequency in EECs.**

*tap^1.3^-B-Gal4>GCaMP6f* flies aged 3 d AE at 25^o^C were fed with 1% L-Glu for 48 h. Guts were then dissected and imaged in LIG using a Zeiss LSM 800 confocal microscope.

**Supplementary Movie 4 | Cytosolic Ca^2+^ oscillation in EECs of *tap^1.3^-B-Gal4>GcaMP6f + attp empty* flies.**

Guts from *tap^1.3^-B-Gal4>GCaMP6f + attp empty* flies aged 3-5 d AE at 25^o^C raised on cornmeal food were dissected and imaged in LIG using a Zeiss LSM 800 confocal microscope.

**Supplementary Movie 5 | Knocking down *stim* in EECs increases Ca^2+^ oscillation frequency.**

Guts from *tap^1.3^-B-Gal4>GCaMP6f + stim^RNAi^* flies aged 3-5 d AE at 25^o^C raised on cornmeal food were dissected and imaged in LIG using a Zeiss LSM 800 confocal microscope.

**Supplementary Movie 6 | Knocking down *SERCA* in EECs reduces Ca^2+^ oscillation frequency.**

Guts from *tap^1.3^-B-Gal4>GCcaMP6f + SERCA^RNAi^* flies aged 3-5 d AE at 25^o^C raised on cornmeal food were dissected and imaged in LIG using a Zeiss LSM 800 confocal microscope.

**Supplementary Movie 7 | Knocking down *PMCA* in EECs reduces Ca^2+^ oscillation frequency.**

Guts from *tap^1.3^-B-Gal4>GCaMP6f + PMCA^RNAi^* flies aged 3-5 d AE at 25^o^C raised on cornmeal food were dissected and imaged in LIG using a Zeiss LSM 800 confocal microscope.
