# Supplementary material for: Dietary L-Glu sensing by enteroendocrine cells adjusts food intake via modulating gut PYY/NPF secretion
Source: Nat Commun. 2024 Apr 25;15:3514. doi: 10.1038/s41467-024-47465-4 (PMC11045819; doi:10.1038/s41467-024-47465-4)
Supplement: Supplementary file 11 — Reporting Summary [file 41467_2024_47465_MOESM11_ESM.pdf]

nature portfolio

Corresponding author(s): Guo, Z.

Last updated by author(s): Mar 20, 2024

Reporting Summary

Nature Portfolio wishes to improve the reproducibility of the work that we publish. This form provides structure for consistency and transparency in reporting. For further information on Nature Portfolio policies, see our Editorial Policies and the Editorial Policy Checklist.

Statistics

For all statistical analyses, confirm that the following items are present in the figure legend, table legend, main text, or Methods section.

n/a	Confirmed	
☐	☒	The exact sample size (*n*) for each experimental group/condition, given as a discrete number and unit of measurement
☐	☒	A statement on whether measurements were taken from distinct samples or whether the same sample was measured repeatedly
☐	☒	The statistical test(s) used AND whether they are one- or two-sided<br>*Only common tests should be described solely by name; describe more complex techniques in the Methods section.*
☒	☐	A description of all covariates tested
☐	☒	A description of any assumptions or corrections, such as tests of normality and adjustment for multiple comparisons
☐	☒	A full description of the statistical parameters including central tendency (e.g. means) or other basic estimates (e.g. regression coefficient) AND variation (e.g. standard deviation) or associated estimates of uncertainty (e.g. confidence intervals)
☐	☒	For null hypothesis testing, the test statistic (e.g. *F*, *t*, *r*) with confidence intervals, effect sizes, degrees of freedom and *P* value noted<br>*Give P values as exact values whenever suitable.*
☒	☐	For Bayesian analysis, information on the choice of priors and Markov chain Monte Carlo settings
☒	☐	For hierarchical and complex designs, identification of the appropriate level for tests and full reporting of outcomes
☒	☐	Estimates of effect sizes (e.g. Cohen's *d*, Pearson's *r*), indicating how they were calculated

Our web collection on statistics for biologists contains articles on many of the points above.

Software and code

Policy information about availability of computer code

Data collection	Zeiss ZEN 2.3 (blue edition) (version 2.3.69.01000) for image acquisition.
Data analysis	ImageJ (version 1.53t)<br>Mathworks MATLAB R2017a (version 9.2.0.518641)<br>GraphPad Prism 8 (version 8.0.1)<br>The information for control circuit and temperature control application had been uploaded to figshare (https://figshare.com/articles/software/Drosophila_local_temperature_control_device/13451204)

For manuscripts utilizing custom algorithms or software that are central to the research but not yet described in published literature, software must be made available to editors and reviewers. We strongly encourage code deposition in a community repository (e.g. GitHub). See the Nature Portfolio guidelines for submitting code & software for further information.

Data

Policy information about availability of data

All manuscripts must include a data availability statement. This statement should provide the following information, where applicable:

- Accession codes, unique identifiers, or web links for publicly available datasets
- A description of any restrictions on data availability
- For clinical datasets or third party data, please ensure that the statement adheres to our policy

All data generated or analyzed during this study are available as Source Data files. Source data are provided with the paper. The gut-microbiota sequencing data generated in this study have been deposited in the Figshare database without accession code [https://doi.org/10.6084/m9.figshare.25458226.v1].

Research involving human participants, their data, or biological material

Policy information about studies with human participants or human data. See also policy information about sex, gender (identity/presentation), and sexual orientation and race, ethnicity and racism.

Reporting on sex and gender	No human studies were performed.
Reporting on race, ethnicity, or other socially relevant groupings	No human studies were performed.
Population characteristics	No human studies were performed.
Recruitment	No human studies were performed.
Ethics oversight	No human studies were performed.

Note that full information on the approval of the study protocol must also be provided in the manuscript.

Field-specific reporting

Please select the one below that is the best fit for your research. If you are not sure, read the appropriate sections before making your selection.

☒ Life sciences ☐ Behavioural & social sciences ☐ Ecological, evolutionary & environmental sciences

For a reference copy of the document with all sections, see nature.com/documents/nr-reporting-summary-flat.pdf

Life sciences study design

All studies must disclose on these points even when the disclosure is negative.

Sample size	Sample size was chosen based on similar previously published studies of Drosophila behavior and metabolism (doi.org: 10.1038/s41467-021-25146-w, doi.org: 10.1038/s41586-020-2866-8, doi.org: 10.1016/j.cmet.2018.09.021, doi.org: 10.1016/j.cmet.2017.01.002, doi.org: 10.1016/j.celrep.2023.112093). No sample-size calculations were performed. The numbers of samples are large enough to capture normal variation while maintaining feasibility for preparation and are similar to or larger than those used in other published studies in the field. qPCR used 3 samples, each containing several tissues or animals, the standard in our lab; food intake measurement and metabolic assays used at least 10 replicates containing multiple animals. Image analyses made use of multiple tissues per genotype or condition, as described in the appropriate figure legends or methods.
Data exclusions	No data were excluded.
Replication	Representative images were chosen from multiple options, generally at least 4. All experiments producing numerical data include at least 3 replicates. All attempts at replication were successful.
Randomization	Animals were randomly grouped into batches as indicated in the text
Blinding	Researchers were not blinded during the study because this is not generally done in fly studies. With limited staff with expertise in these particular studies, the person handling sample prep must usually also be the one performing the assay.

Reporting for specific materials, systems and methods

We require information from authors about some types of materials, experimental systems and methods used in many studies. Here, indicate whether each material, system or method listed is relevant to your study. If you are not sure if a list item applies to your research, read the appropriate section before selecting a response.

Materials & experimental systems

n/a	Involved in the study
	[x] Antibodies
[x]	Eukaryotic cell lines
[x]	Palaeontology and archaeology
	[x] Animals and other organisms
[x]	Clinical data
[x]	Dual use research of concern
[x]	Plants

Methods

n/a	Involved in the study
[x]	ChIP-seq
[x]	Flow cytometry
[x]	MRI-based neuroimaging

Antibodies

Antibodies used

Chicken anti-GFP, 1:10,000, Abcam #AB13970.
Rabbit anti-RFP, 1:10000, Abcam #62341.
Mouse anti-Pros, 1:200, Developmental Studies Hybridoma Bank #528440.
Mouse anti-NC82, 1:100, Developmental Studies Hybridoma Bank #2314866.
Rabbit anti-LacZ, 1:4000, CUSABIO #CSB-PA009476LA01ENV.
Rabbit anti-PH3, 1:10000, Millipore #MMI-06-570.
Mouse anti-NPF, 1:200, obtained from Veenstra, J. A. (University of Bordeaux).
Rabbit anti-NPF, 1:4000, this paper.
Rabbit anti-Tk, 1:4000, obtained from Benjamin Ohlstein (University of Texas Southwestern Medical Center).
Rabbit anti-HA, 1:4000, Cell Signaling Technology #3724S.
Rabbit anti-NPFR, 1:2000, RayBiotech #RB-19-0003-200.
Rabbit anti-AKH, 1:10000, obtained from Wei Song (Wuhan University).
Rabbit anti-TH, 1:4000, Abcam #AB112.
Alexa Flour goat anti-chicken 488, 1:4000, Invitrogen #A11039.
Alexa Flour goat anti-rabbit 488, 1:4000, Invitrogen #A11008.
Alexa Flour goat anti-rabbit 555, 1:4000, Invitrogen #A21428.
Alexa Flour goat anti-mouse 555, 1:4000, Invitrogen #A21422.
Alexa Flour goat anti-mouse 647, 1:4000, Invitrogen #A21235.

Validation

Chicken anti-GFP (Abcam #AB13970) (https://www.abcam.com/products/primary-antibodies/gfp-antibody-ab13970.html)
Rabbit anti-RFP (Abcam #62341) (https://www.abcam.com/products/primary-antibodies/rfp-antibody-ab62341.html)
Mouse anti-Pros (Developmental Studies Hybridoma Bank #528440) (https://dshb.biology.uiowa.edu/Prospero-MR1A)
Mouse anti-NC82 (Developmental Studies Hybridoma Bank #2314866) (https://dshb.biology.uiowa.edu/nc82)
Rabbit anti-LacZ (CUSABIO #CSB-PA009476LA01ENV) (https://www.cusabio.com/Polyclonal-Antibody/lacZ-Antibody-12783205.html)
Rabbit anti-PH3 (Millipore #MMI-06-570) (https://www.merckmillipore.com/CN/zh/product/Anti-phospho-Histone-H3-Ser10-Antibody-Mitosis-Marker,MM_NF-06-570)
Mouse anti-NPF validated in Veenstra, J.A., Agricola, H.J. & Sellami, A. Regulatory peptides in fruit fly midgut. Cell and tissue research 334, 499-516 (2008).
Rabbit anti-Tk validated in Ohlstein, B. & Spradling, A. The adult Drosophila posterior midgut is maintained by pluripotent stem cells. Nature 439, 470-474 (2006).
Rabbit anti-HA (Cell Signaling Technology #3724S) (https://www.cellsignal.com/products/primary-antibodies/ha-tag-c29f4-rabbit-mab/3724)
Rabbit anti-NPFR (RayBiotech #RB-19-0003-200) (https://www.raybiotech.com/rabbit-anti-npf-receptor-n-terminus-rb-19-0003)
Rabbit anti-AKH validated in Li, Y.G. et al. Gut AstA mediates sleep deprivation-induced energy wasting in Drosophila. Cell Discov 9 (2023).
Rabbit anti-TH (Abcam #AB112) (https://www.abcam.com/products/primary-antibodies/tyrosine-hydroxylase-antibody-neuronal-marker-ab112.html)

Animals and other research organisms

Policy information about studies involving animals; ARRIVE guidelines recommended for reporting animal research, and Sex and Gender in Research

Laboratory animals

This study made use of a variety of stocks of Drosophila melanogaster, detailed in the manuscript and Supplementary Table 1. 2-10 day-old adult female flies were used in this study. Detailed information is provided in Methods.

Stocks created for this work:
NPF-0.7-GFP
NPFR::3XHA
tap1.3-A-Gal4
tap1.3-B-Gal4

Stocks obtained from Bloomington Drosophila stock center:

esg-Gal4, #93857
UAS-attp2 empty, #36303
UAS-attp40 empty, #36304
canton-s, #64349
tap1.3-Gal4, #46377
UAS-nls-GFP, #4776
UAS-clumsy RNAi, #28351
vm-Gal4, #48547
How-Gal4, #1767
GMR60E02-Gal4, #39250
GMR60G05-Gal4, #39259
GMR61H06-Gal4, #39281
GMR65C12-Gal4 , #39348
20XUAS-6xGFP, #52262
UAS-shi ts, #66600
Ddc-LexA, #54218

Stock obtained from Vienna Drosophila Resource Center:
w1118, #60000

Stocks obtained from TsingHua Fly Center:
UAS-scute RNAi, #2205
UAS-NPF RNAi, #2569
UAS-Tk RNAi, #2022
UAS-stim RNAi, #2581
UAS-SERCA RNAi, #2107
UAS-PMCA RNAi, #1887
UAS-IP3R RNAi, #02220.N
UAS-CG11155 RNAi, #3285
UAS-Ekar RNAi, #3080
UAS-GluRIA RNAi 1, #201500449.S
UAS-GluRIA RNAi 2, #2683
UAS-GluRIA RNAi 3, #5238
UAS-GluRIB RNAi 1, #2758
UAS-GluRIB RNAi 2, #5273
UAS-GluRIB RNAi 3, #5358
UAS-GluRIIA RNAi, #2659
UAS-GluRIIB RNAi, #3089
UAS-GluRIIC RNAi, #2049
UAS-GluRIID RNAi, #2151
UAS-GluRIIE RNAi, #3986
UAS-Grik RNAi, #3979
UAS-KaiR1D RNAi, #3982
UAS-mGluR RNAi 1, #5288
UAS-mGluR RNAi 2, #2115
UAS-mtt RNAi 1, #0827
UAS-mtt RNAi 2, #5594
UAS-Nmdar1 RNAi 1, #2118
UAS-Nmdar1 RNAi 2, #5286
UAS-Nmdar1 RNAi 3, #5287
UAS-Nmdar2 RNAi 1, #5240
UAS-Nmdar2 RNAi 2, #5249
UAS-Nmdar2 RNAi 3, #5862
UAS-NPFR RNAi, #2116
UAS-ChAT RNAi 1, #02505.N
UAS-ChAT RNAi 2, #201500313.S
UAS-Ddc RNAi, #2416
UAS-Vmat RNAi, #01473.N
UAS-Gad RNAi 1, #02214.N
UAS-Gad RNAi 2, #201500431.S
UAS-VGAT RNAi, #4304
UAS-Hdc RNAi, #2140
UAS-Tbh RNAi 1, #02221.N
UAS-Tbh RNAi 2, #201500898.S
UAS-Tdc2 RNAi, #2075
UAS-Trh RNAi, #2276
UAS-VGlut RNAi, #2700

Others:
NRE-LacZ, esg-Gal4, tub-Gal80 ts, UAS-GFP -- gift of Benjamin Ohlestin, University of Texas Southwestern Medical Center
UAS-hid -- gift of Benjamin Ohlestin, University of Texas Southwestern Medical Center
OreR -- gift of Jianhua Huang, Zhejiang University
Tkg-Gal4 -- gift of Wei Song, Wuhan University
UAS-TNT-imp -- gift of Zhihua Liu, Hubei University
UAS-TNT-G2 -- gift of Zhihua Liu, Hubei University
UAS-TrpA1 -- gift of Yufeng Pan, Southeast University
UAS-Denmark,UAS-nsyt:GFP -- gift of Yufeng Pan, Southeast University
13XLexAop-myr:GFP, UAS-mCD8:RFP;;10XUAS-CaLexA -- gift of Yufeng Pan, Southeast University
8xLexAop-FlpL,UAS>stop>myr:GFP -- gift of Yufeng Pan, Southeast University
trans-tango -- gift of Yufeng Pan, Southeast University
13xLexAop-myr:GFP,UAS-mCD8:RFP -- gift of Yufeng Pan, Southeast University
NPFR-RA/C-Gal4 -- gift of Yi Rao, Peking University
NPFR-RB/D-Gal4 -- gift of Yi Rao, Peking University
NPFR-RA/C-LexA -- gift of Yi Rao, Peking University
NPF attP (NPF null) -- gift of Yi Rao, Peking University
NPFR attP (NPFR null) -- gift of Yi Rao, Peking University
Pros v1-Gal4,tub-Gal80 ts,UAS-GFP -- gift of Jean-François Ferveur, Université Paris-Sud
UAS-pANF-EMD -- gift of David Deitcher, Cornell University
NPF sk1 (NPF 1) -- gift of Shu Kondo, Tokyo University of Science
NPFR 8 -- gift of Shu Kondo, Tokyo University of Science
UAS-GcaMP6f -- gift of Shan Jin, Hubei University
UAS-tdTomato -- gift of Kenneth Irvine, Rutgers University
UAS-NPF -- gift of Todd Schlenke, University of Arizona
UAS-mCD8:GFP, UAS-Redstinger -- gift of Woo Jae Kim, HIT Center for Life Sciences, HIT

Wild animals: No wild animals were used in this study.

Reporting on sex: All experiments were performed with mated female animals.

Field-collected samples: No field-collected animals were used in this study.

Ethics oversight: No ethics approval or oversight is required for Drosophila studies.

Note that full information on the approval of the study protocol must also be provided in the manuscript.

Plants

Seed stocks: *Report on the source of all seed stocks or other plant material used. If applicable, state the seed stock centre and catalogue number. If plant specimens were collected from the field, describe the collection location, date and sampling procedures.*

Novel plant genotypes: *Describe the methods by which all novel plant genotypes were produced. This includes those generated by transgenic approaches, gene editing, chemical/radiation-based mutagenesis and hybridization. For transgenic lines, describe the transformation method, the number of independent lines analyzed and the generation upon which experiments were performed. For gene-edited lines, describe the editor used, the endogenous sequence targeted for editing, the targeting guide RNA sequence (if applicable) and how the editor was applied.*

Authentication: *Describe any authentication procedures for each seed stock used or novel genotype generated. Describe any experiments used to assess the effect of a mutation and, where applicable, how potential secondary effects (e.g. second site T-DNA insertions, mosiacism, off-target gene editing) were examined.*
